# Clinical Assessment of the Ambu® Aura-i™ as an Independent Ventilatory Device and Conduit for Fiberoptic-Guided Endotracheal Intubation in Adults

**DOI:** 10.7759/cureus.58629

**Published:** 2024-04-20

**Authors:** Navya Mishra, Nitesh Sinha, Ramesh K Kharwar, Jay Prakash

**Affiliations:** 1 Anesthesiology, Rajendra Institute of Medical Sciences (RIMS), Ranchi, IND; 2 Anesthesiology and Critical Care, Rajendra Institute of Medical Sciences (RIMS), Ranchi, IND

**Keywords:** ventilation device, supraglottic airway device, intubation, fiber optic, ambu aura-i

## Abstract

Background and aim

A novel supraglottic airway device of the second generation is the Ambu® Aura-i™. It is designed to accommodate standard cuffed tracheal tubes and is phthalate-free and compatible with MRI. The primary objectives of the research were to examine the properties and efficiency of Ambu® Aura-i™ as a means of enabling fiberoptic-guided intubation, the view of the glottis during fiber optic examination, the duration of intubation in fiber optic bronchoscopy, the ease of intubation, the success rate of intubation, and the duration for device removal from the tracheal tube.

Methodology

A hospital-based descriptive observational study was conducted with 80 patients. An adequately sized Ambu® Aura-i™ was placed after general anesthesia was induced. Following a fiberoptic examination of the view of the glottis through the Ambu® Aura-i™, the trachea was intubated under fiberoptic guidance. The Ambu® Aura-i™ insertion time, glottic view grading, ease of intubation, and time required for fiberoptic-guided intubation were recorded. Also, the time taken to remove the Ambu® Aura-i™ was documented.

Results

Similar levels of ease were experienced by both groups after inserting the Ambu® Aura-i™, being easy in both group 1 (37/40) and in group 2 (38/40). In group 1, the average time taken to insert the Ambu® Aura-i™ was 13.53±1.91 seconds, while in group 2, it was 13.98±2.4 seconds. The average time required for fiberoptic-guided intubation was found to be 14.95±1.85 seconds in group 1 and 14.15±1.37 seconds in group 2, indicating a statistically negligible variation.

Conclusion

The low cost of Ambu® Aura-i™, size suitability and availability for almost all age groups, compatibility with MRI machines, and availability in phthalate-free versions contribute to it being a more appealing and useful ventilatory device, as well as an intubation tool for both normal and emergency airway management.

## Introduction

Keeping the airway patent is the fundamental goal of an anesthesiologist. The invention of the laryngeal mask airway by Dr. Archie Brain in 1981 completely changed the way airways were managed during anesthesia [1,2]. Since then, many additions and improvisations have been made to supraglottic airway devices. Among them, the Ambu® Aura-i™, a second-generation supraglottic airway device manufactured by Royal Surgical Company, Mumbai, India, stands out [3].

The primary objective of the research was to examine the features and efficacy of the Ambu® Aura-i™ as a means of enabling fiberoptic-guided intubation. Specifically, the study focused on observing and recording key parameters, including the glottic view during fiberoptic examination, the time required for guided intubation through a fiberscope (in seconds), the ease of intubation, the number of attempts required for successful intubation, and the time required to remove the device from over the tracheal tube (in seconds).

## Materials and methods

This hospital-based descriptive observational study was conducted at MGM Hospital, Jaipur, from January 2017 to June 2018, following ethical committee clearance (IEC JPC 323 dated 03.03.2017). Prior written informed consent was obtained from 80 consenting adult patients (American Society of Anesthesiologists (ASA) I and II) who fulfilled all criteria for undergoing elective surgery requiring tracheal intubation. Based on comparable earlier studies [4,5,6,7], the sample size was determined to be 80, taking into account the predicted average time required for inserting the Ambu® Aura-i™ sizes 3 and 4, with a power of 80% and a confidence interval of 95%.

All patients undergoing tracheal intubation for elective surgery and anesthesia were eligible for the study if they were aged between 18 and 55 years, weighed between 30 to 70 kg, regardless of gender, had a mouth opening of more than 2.5 cm, and did not have a predicted difficult airway.

Exclusion criteria included intraoral pathology, cardiovascular or respiratory disorders, and contraindications to Laryngeal Mask Airways (LMA) including reflux esophagitis. Patients unwilling to provide consent were also excluded.

Study protocol

Patients were stratified into two groups based on body weight, and the appropriately sized Ambu® Aura-i™ was selected for each group. Group 1 (30-50 kg) used Ambu® Aura-i™ (size 3), while Group 2 (50-70 kg) used Ambu® Aura-i™ (size 4). Each group consisted of forty individuals.

To achieve complete pre-oxygenation, 100% oxygen was administered using a facemask for three minutes. Anaesthesia was induced with IV propofol at 2.5 mg/kg. Thereafter, the Ambu® Aura-i™ was introduced following jaw relaxation.

Assessment of study parameters

Ambu® Aura-i™ Insertion

After applying a jaw thrust to patients in both groups, a device of the relevant size (depending on the patient's body weight) was inserted using the manufacturer’s suggested standard midline technique. Thirty milliliters of air were used to inflate the cuff after implantation. The correct placement of the Ambu® Aura-i™ was confirmed by a rectangular curve on the monitor, which showed a normal capnogram (ETCO2 > 30 mm Hg).

Time taken to insert Ambu® Aura-i™ : The time taken to insert the Ambu® Aura-i™ was measured from the moment the introducer anesthetist picked up the device to the moment the first capnographic waveform appeared after insertion. Extension, flexion, and Chandy's maneuver were performed in order to correct the placement if any signs of resistance, leakage, or audible leaks were observed during the procedure. The appearance of an unusual capnograph (non-rectangular with an EtCO2 below 30 mmHg) was treated similarly.

Failure to introduce the airway: A maximum of three attempts were allowed. If, after three attempts, the patient's oxygen saturation dropped below 92%, or if a capnogram was not present, the patient was intubated using conventional laryngoscopy.

Following the correct implantation of the Ambu® Aura-i™, anesthesia was deepened with intravenous atracurium (0.6 mg/kg) and a gaseous mixture of oxygen, nitrous oxide, and isoflurane at 1.2%.

Fiberoptic Evaluation of Glottic View

This was performed three minutes after administering atracurium. Using a Sortz flexible intubating fiberscope, preloaded with a cuffed, lubricated, flexometallic tracheal tube (7.0/7.5 mm in diameter) without a connector, an independent investigator evaluated the view of the glottis. This was done while keeping the primary investigator blind to the grade and view of the glottis. The independent investigator executed all necessary maneuvers to enhance the glottis view using fiberoptic vision and recorded their findings in Table 1.

**Table 1:** Fibreoptic glottic view grading. Table included from Reference [8], and permission was prior obtained from the authors of the article.

Grade	Fibreoptic View
1	Visible larynx only
2	Larynx and epiglottis posterior surface seen
3	Larynx and epiglottis tip of anterior surface visible,<50% visual obstruction of epiglottis to larynx.
4	Epiglottis downfolded, anterior surface visible,>50% visual obstruction of epiglottis to larynx.
5	Epiglottis downfolded and larynx not visible

Fiberoptic-Guided Intubation Through Ambu® Aura-i™

After obtaining the best view of the glottic aperture, a preloaded tracheal tube was used for fiberoptic-guided tracheal intubation. After reconnecting the tracheal tube along with its connector, further ventilation using nitrous oxide, oxygen, and isoflurane was carried out. If the Ambu® Aura-i™ intubation method failed, tracheal intubation with direct laryngoscopy was performed in the supine position.

The intubation time with the fiberscope was recorded as the time elapsed between the insertion of the bronchoscope into the open end of the Ambu® Aura-i™ and the first appearance of the capnogram waveform after successful tube placement.

Removal of Ambu® Aura-i™ Over the Tracheal Tube

Following the verification of successful intubation, the Ambu® Aura-i™ was removed using a smaller size 6mm tracheal tube. The in situ tracheal tube was held in place with the other hand to avoid inadvertent extubation. A repeat check for a normal capnogram, as obtained after successful endotracheal intubation, was conducted, and the tube was secured with tube ties. The removal of the Ambu® Aura-i™ marked the concluding endpoint of the study.

The duration required to effectively remove the Ambu® Aura-i™ was quantified as the time between turning ventilation off and back on following the insertion of the endotracheal tube.

For intraoperative care, a standardized anesthetic regimen containing nitrous oxide, oxygen, isoflurane, atracurium, and fentanyl was used. At the conclusion of the procedure, the neuromuscular block was reversed, and the patient was extubated once the necessary reversal conditions were met.

Data recorded for comparison between the groups included the time duration required to insert the Ambu® Aura-i™ and install the tracheal tube, the number of attempts, the difficulty faced during device insertion, the number and type of adjustment operations, and any complications.

A two-tailed independent t-test was employed for the statistical study between the two groups. Descriptive data within groups were analyzed using a paired t-test. For the study of categorical data, Fisher's exact probability test was utilized. A significance level of P ≤ 0.05 was adopted.

## Results

Ambu® Aura-i™'s ease of insertion is shown in group 1 (Table 2).

**Table 2:** Ease of insertion of Ambu® Aura-i™.

	Group I	Group 2	
	No	No	P-value
Easy	37	38	0.578
Difficult	3	2	

The time it took to insert the Ambu® Aura-i™ in Group 1 was, on average, 13.53 ± 1.91 seconds, while in Group 2, it was 13.98 ± 2.4 seconds (Table 3).

**Table 3:** Time taken in seconds for insertion of Ambu® Aura-i™.

	Group 1	Group 2	
Time Taken in Seconds	Mean± SD	Mean±SD	P-value
	13.53±1.91	13.98±2.41	0.563

Case distribution based on the number of Ambu® Aura-i™ insertion attempts

In Group 1, the insertion of Ambu® Aura-i™ was completed on the first attempt for 33 out of 40 patients (82.5%), two attempts were needed for 5 patients (12.5%), and three attempts were required for the remaining 2 patients (5%). In Group 2, 35 out of 40 patients (87.5%) had the Ambu® Aura-i™ placed on the first attempt; 4 patients (10%) needed two attempts; and 1 patient (2.5%) required three attempts (Table 4).

**Table 4:** Distribution of the cases according to the number of attempts of insertion of Ambu® Aura-i™.

No. of attempts	Group 1		Group 2		
	No.	%	No.	%	P-value
1	33	82.5	35	87.5	0.059
2	5	12.5	4	10	
3	2	5	1	2.5	
Total	40	100	40	100	

Grades of Glottis

A fiber optic scope was used to assess glottic vision grading in Group 1 and Group 2. Group 1 comprised 80% of patients with a grade of 1, whereas Group 2 had 87.5% of patients with the same grade. A Grade 2 view was present in 20% of patients in Group 1 and 12.5% of participants in Group 2. There was no statistical difference in the glottic vision scores between either group (Table 5).

**Table 5:** Table showing fiberoptic assessment of grading of glottic view in Group 1 and Group 2.

Fiberoptic Intubation	Group 1		Group 2		P-value
Grade of glottis	No.	%	No.	%	
Grade 1	32	80	35	87.5	0.567
Grade 2	8	20	5	12.5	
Grade 3	0	0	0	0	
Grade 4	0	0	0	0	
Grade 5	0	0	0	0	

Groups 1 and 2 had significantly different mean times for intubation (via the fiberscope), with Group 1 taking 14.95 ± 1.85 seconds and Group 2 taking 14.15 ± 1.37 seconds (Table 6). However, these differences were not statistically significant.

**Table 6:** Time taken in fiberoptic-guided intubation.

	Group 1		Group 2		
	Mean	SD	Mean	SD	P-value
Time taken in seconds	14.95	1.85	14.15	1.37	0.054

The number of attempts made by each group did not show any discernible differences. The percentage of people who were successfully inserted with the device on the first try was 75% in Group 1 and 87.5% in Group 2 (Table 7).

**Table 7:** Number of attempts for fiberoptic-guided intubation.

Number of Attempts	Group 1		Group 2		P-value
	No.	%	No.	%	0.332
1	30	75	35	87.5	
2	7	17.5	4	10	
3	3	7.5	1	2.5	

Group 1 took an average of 11.87 ± 1.265 seconds to remove the mask, and Group 2 took an average of 11.56 ± 1.457 seconds to railroad the mask over the tracheal tube (Table 8).

**Table 8:** Distribution of the cases according to time taken in removal of Ambu® Aura-i™.

	Group 1		Group 2		P-value
	Mean	SD	Mean	SD	
Time taken (in sec) in removal of Ambu® Aura-i™	11.87	1.265	11.25	1.58	0.054

## Discussion

In all 80 patients, or 100% of the cohort, we were able to effectively implant the Ambu® Aura-i™. Among them, 83% of patients had their intubation completed on the first attempt, 12% on the second attempt, and 5% on the third. Yahaya Z et al. [4] and de Lloyd LJ et al. [5] reported similar observations in their studies.

In our study, of the 40 patients in Group 1, 37 experienced easy placement of the Ambu® Aura-i™, and we encountered some difficulty in 3 cases. Of the 40 patients in Group 2 who were intubated using the Ambu® Aura-i™, 38 had easy placement, and only two faced difficulties with insertion. Overall, the difference was not statistically significant, mirroring observations reported in previous studies [4,6-9]. The mean time taken for Ambu® Aura-i™ insertion was 13.53±1.91 seconds in Group 1 and 13.98±2.4 seconds in Group 2, showing no significant difference. Similar findings were reported in previous studies [6,7].

In our study, the glottic view was classified as Grade 1 in 80% of patients in Group 1, while 92% of patients in Group 2 had a Grade 1 glottis view, with little discernible statistical difference. Yahaya Z et al. also reported similar findings [4]. Regarding the number of attempts made in each group to intubate with the Ambu® Aura-i™, no statistically significant difference was observed. On the first try, 75% in Group 1 and 87.5% in Group 2 were successfully intubated, as recorded in previous studies [6,7]. The mean fiberoptic-guided intubation time was 14.95±1.85 seconds in Group 1 and 14.15±1.37 seconds in Group 2. The difference in times was statistically low and insignificant, in line with other investigations [4,8-12]. Group 1 took approximately 11.87 ± 1.265 seconds to remove the Ambu® Aura-i™, whereas Group 2 took 11.25±1.58 seconds, with removal times being comparable. Similar observations were made by Rangaswamy TM et al. [6]. We found that neither study group experienced any significant adverse events, such as aspiration, laryngospasm, unintentional extubation, oxygen desaturation, or esophageal intubation, unlike what was reported in some previous studies [9,12,13].

A limitation of our study was that only sizes 3 and 4 of the Ambu® Aura-i™ were available in our institute, thereby restricting our patient inclusion to those weighing up to 70 kgs

## Conclusions

When attempting to establish and maintain control of the airway during normal or emergency anesthetic operations, the Ambu® Aura-i™ is an excellent substitute for a face mask in patients evaluated as eligible for a supraglottic airway, or in situations where other methods of airway establishment have failed. For the purpose of establishing an airway, the Ambu® Aura-i™ is the recommended device in cases where an endotracheal tube (ETT) is not considered necessary, or when unforeseen challenges related to airway management occur.

Tracheal intubation with the Ambu® Aura-i™ airway tube is easier due to its curved, broader, and more rigid design. The airway tube’s flexible cuff and stiff connector facilitate easy and painless insertion and removal. Offered in several sizes based on patients' body weight, which ranges from less than 5 kg to more than 100 kg, it accommodates a wide spectrum of patients.

The Ambu® Aura-i™ is a cost-effective ventilatory device and intubation tool, making it both anesthetist- and patient-friendly. Its sizes are suitable for all age groups, and it is compatible with multiple radiography machines. Additionally, its phthalate-free version is currently available. In the upcoming years, the Ambu® Aura-i™ might prove to be of great use in fiberoptic-guided airway management, both in the operation theater and in the ICU.
